# Comparison of Clinical Performance Between Digital Breast Tomosynthesis and MammouS-N

**DOI:** 10.3390/tomography12020017

**Published:** 2026-01-30

**Authors:** Sung Ui Shin, Mijung Jang, Bo La Yun, Su Min Cho, Yoon Yeong Choi, Bohyoung Kim, Min Jung Kim, Sun Mi Kim

**Affiliations:** 1 Department of Radiology, Seoul National University Bundang Hospital, Seoul National University College of Medicine, 82, Gumi-ro 173 Beon-gil, Bundang-gu, Seongnam-si 13620, Gyeonggi-do, Republic of Korea; shinsungui@gmail.com (S.U.S.); mjjang74@gmail.com (M.J.); y2choi822@gmail.com (Y.Y.C.); 2 Department of Biomedical Engineering, Hankuk University of Foreign Studies, Yongin-si 17035, Gyeonggi-do, Republic of Korea; 3 Department of Radiology, Severance Hospital, Research Institute of Radiological Science, Yonsei University College of Medicine, Seoul 03722, Republic of Korea

**Keywords:** breast, ultrasonography, mammography

## Abstract

**Background/Objectives**: We compared the visibility of breast cancer using the newly developed standing automated breast ultrasound system (MammouS-N) and digital breast tomosynthesis (DBT), and identified factors influencing lesion visibility. **Methods**: We prospectively enrolled 100 women (mean age: 51.6 years; range: 26–76 years) who were diagnosed with breast cancer and were scheduled to undergo DBT between January and July 2024. They underwent DBT and an ultrasound on the same day. Two radiologists evaluated the visibility scores (0–5) of lesions corresponding to biopsy-confirmed breast cancers identified using magnetic resonance imaging. The Wilcoxon signed-rank test was used to compare the visibility scores of cancers identified on DBT and/or MammouS-N images. **Results**: Among the 100 women, invasive ductal carcinoma was the most common malignancy (73%). DBT findings included negative findings (7%), masses (46%), masses with calcification (29%), calcifications only (15%), and architectural distortions (3%). On MammouS-N ultrasound, most lesions were classified as masses (93%), whereas 7% were non-mass lesions. For Reviewer 1, MammouS-N demonstrated significantly higher visibility scores (higher scores: 26 on MammouS-N, seven on DBT; equal scores: 67, z = −3.234, *p* = 0.001). For Reviewer 2, the two modalities showed no significant difference in visibility (higher scores: 27 on MammouS-N, 28 on DBT, equal scores: 45, z = −0.040, *p* = 0.968). Noncalcified lesions that were obscured on DBT were better visualized on MammouS-N (*p* < 0.001) by both reviewers. **Conclusions**: MammouS-N holds promise as an imaging modality complementary to DBT in women with dense breast tissue, particularly for non-calcified lesion detection.

## Simple Summary

Dense breast tissue complicates breast cancer detection using mammography alone. This study compared digital breast tomosynthesis with a novel standing automated breast ultrasound system in women with biopsy-confirmed breast cancer. Tomosynthesis effectively detected calcified lesions, while ultrasound provided superior visualization of non-calcified lesions without radiation exposure. The ultrasound system offers automated and reproducible imaging that integrates seamlessly into existing workflows, making it a valuable complement to tomosynthesis, especially for women with dense breasts. These findings support further studies on combined imaging approaches to improve breast cancer screening and diagnosis.

## 1. Introduction

Advancements in imaging technology have considerably improved breast cancer screening and diagnosis. With the increasing global incidence of breast cancer, there is a growing need for ongoing evaluation of diagnostic methods to ensure early and accurate detection [1].

Digital breast tomosynthesis (DBT), also known as 3D mammography, offers notable advantages by providing three-dimensional breast tissue views, effectively overcoming the tissue overlap limitations of standard mammograms and improving lesion detection [2]. However, DBT has notable drawbacks. It delivers higher ionizing radiation doses than conventional mammography, raising safety concerns for patients requiring frequent screenings [2,3]. It is also more expensive and less accessible than traditional mammography or ultrasound, potentially restricting patient access. Although DBT enhances cancer detection in dense breast tissue, it may fail to identify certain lesions detectable by ultrasound, particularly in women with extremely dense breast tissue [4].

Traditional handheld breast ultrasound offers several advantages over DBT, including complete radiation-free imaging, eliminating the safety concerns associated with the higher ionizing radiation doses of DBT. Ultrasound is particularly effective for evaluating dense breast tissue, while DBT may still miss certain lesions. Ultrasound also provides real-time imaging capabilities that DBT cannot offer, enabling immediate lesion assessment and image-guided biopsies [5]. However, handheld breast ultrasound has certain limitations. Image quality can vary considerably depending on the operator’s skill and experience, introducing variability that may affect diagnostic consistency. Additionally, handheld breast ultrasound may miss lesions that are not included in the scanned area, particularly if the examination is not thoroughly performed across the entire breast [6].

The standing automated breast ultrasound system (ABUS), MammouS-N (Medicalpark, Yongin, Republic of Korea), is a recently introduced imaging modality that employs ultrasound technology to generate images similar in appearance to those produced by DBT. This technique offers certain advantages over DBT and handheld ultrasound. Particularly, it avoids ionizing radiation, thereby resolving the safety concerns associated with DBT. MammouS-N is a standing automated breast ultrasound system designed to acquire volumetric images of the entire breast in three orthogonal planes. This systematic approach aims to facilitate standardized and comprehensive breast coverage, addressing some of the inherent operator-dependence and variability associated with conventional handheld ultrasound. With these features, MammouS-N may contribute to improved accuracy and accessibility of breast imaging. This study aimed to compare DBT and MammouS-N in terms of breast cancer visibility and to identify the factors influencing lesion visibility.

## 2. Materials and Methods

### 2.1. Study Population

This prospective study was approved by the institutional review board, and written informed consent was obtained from all participants. We enrolled 100 patients (mean age: 51.6 years; range: 26–76 years) who were recently diagnosed with breast cancer and were scheduled to undergo DBT between January and July 2024. All patients were referred to our tertiary center for further evaluation and surgery. Of these, 53% were asymptomatic patients whose lesions were initially detected through screening at outside clinics. Pre-referral diagnosis was established via ultrasound-guided biopsy (*n* = 99) or mammography-guided biopsy (*n* = 1). We excluded women with a history of breast surgery, those with contraindications to mammography (pregnancy or lactation), and those without pathological confirmation of breast malignancy. DBT, handheld breast ultrasound, and MammouS-N images were acquired on the same day, and breast magnetic resonance imaging (MRI) was performed within 2 weeks. After the MammouS-N scan, each patient was asked to rate their pain experience as none, mild, moderate, or severe, and to compare it with their experience during DBT. Of the 100 patients, 3 did not undergo surgical treatment, and 14 received neoadjuvant systemic therapy prior to surgery.

### 2.2. DBT

All patients underwent consecutive DBT and conventional full-field digital mammography using single-breast compression (combo mode). All DBT and combo-mode images (bilateral craniocaudal [CC] and mediolateral oblique [MLO] projections) were acquired using one of two DBT systems: the Selenia Dimensions System (Hologic, Bedford, MA, USA) or Senographe Pristina (GE Medical Systems, Buc, France). For each breast, images were obtained for both CC and MLO projections.

### 2.3. MRI

MRI was used to evaluate the location and size of the lesions. All patients underwent MRI to check for multifocal, multicentric, or contralateral breast cancer. Examinations were performed using a 3T MRI system (Achieva or Ingenia; Philips Medical Systems, Best, The Netherlands) equipped with a dedicated breast coil contrast enhancement.

### 2.4. MammouS-N

**Figure 1:**
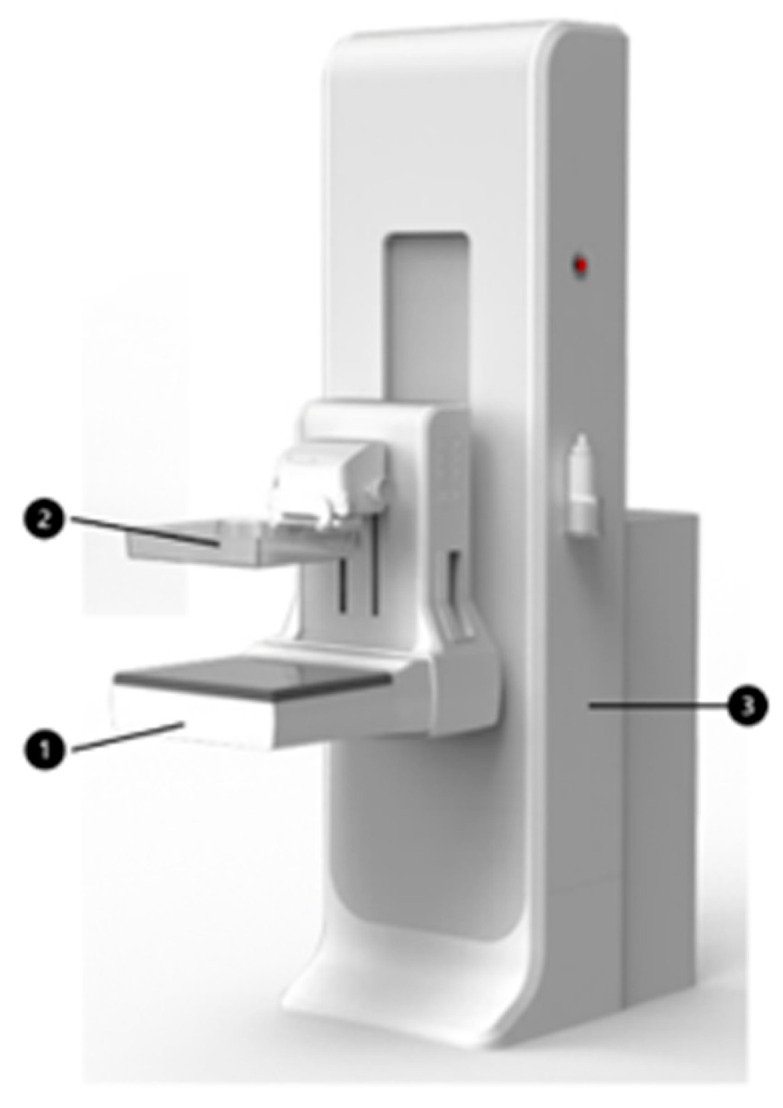
Overview of the MammouS-N system. 1. Breast Ultrasound Scanner: Captures ultrasound images of the breast. 2. Breast Compression Device: Applies compression to the breast during imaging for improved contact and stability. 3. Device Stabilization Structure: provides structural support and stability for the entire system.

**Figure 2:**
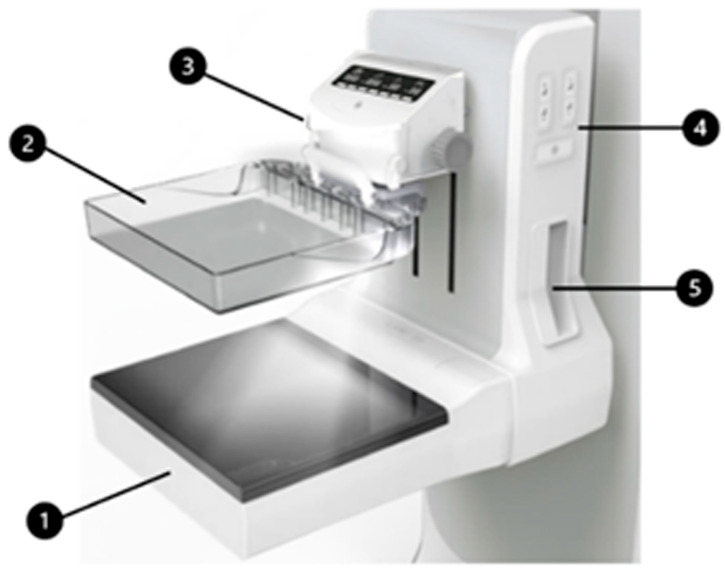
Main imaging components of the MammouS-N system. 1. Breast Ultrasound Scanner: Automatically captures images across the compressed breast surface. 2. Breast Compression Device: A compression plate that ensures firm contact between the breast and the ultrasound probe. 3. Manual Compression Adjustment Handle: Allows manual raising and lowering of the compression device. 4. Breast Stabilization Device Control Console: Control panel for operating the breast stabilization system. 5. Patient Stabilization Handle: A support handle for the patient to hold during the imaging process.

All MammouS-N assessments were performed by one of three radiology technologists, each extensively trained in both ABUS and mammography, using the MammouS-N system (Medicalpark, Yongin, Republic of Korea). The MammouS-N scan was fully automated and continuous, utilizing a 5–12 MHz wide-aperture linear probe to cover an area of up to 30 × 22 cm, with a maximum penetration depth of 8 cm (Figure 1 and Figure 2). Each scan generated approximately 800 slices per volume at an 8 cm depth and 1280 slices at a 2 cm depth.

**Figure 3:**
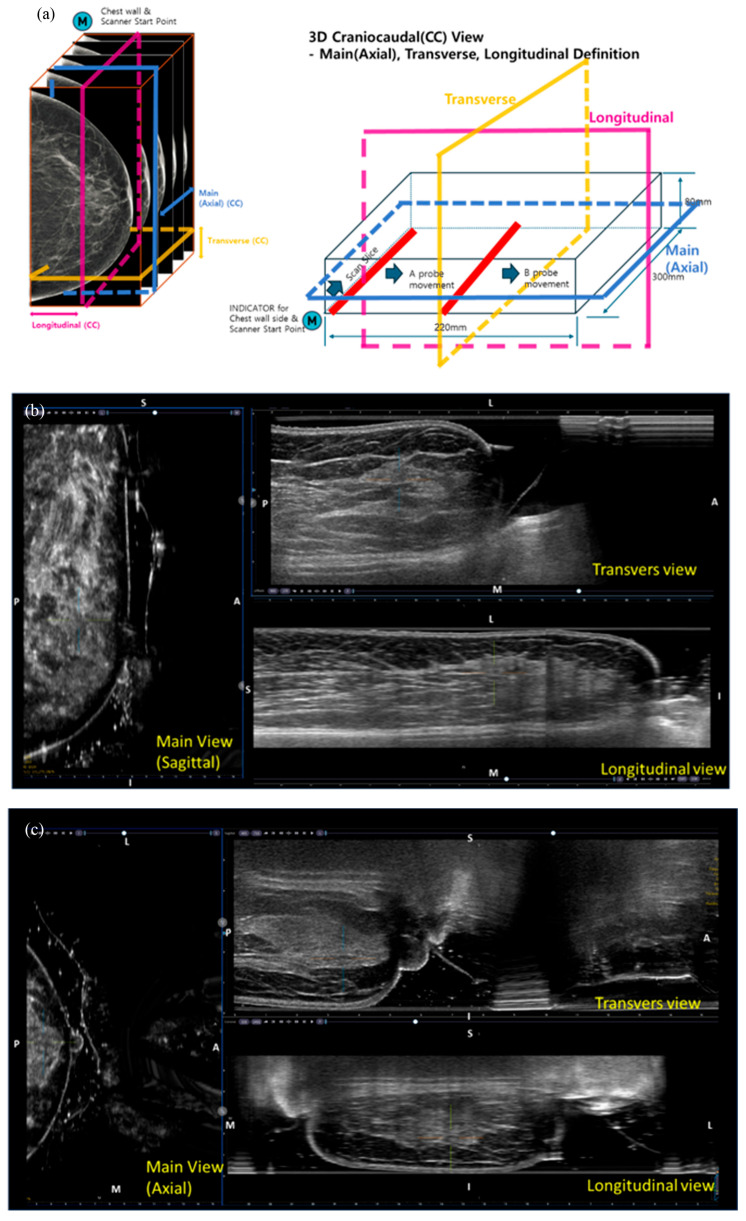

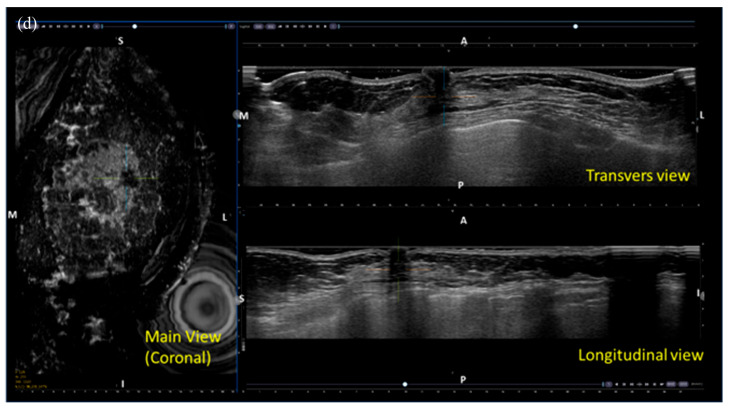
Images of the MammouS-N system: (**a**) Multiplanar views (axial, transverse, and longitudinal) corresponding to mediolateral, craniocaudal, and posteroanterior volumes. (**b**) Mediolateral oblique view: The left image shows the sagittal view corresponding to a mammographic mediolateral oblique projection. The upper-right and lower-right images represent transverse and longitudinal views, respectively. (**c**) Craniocaudal view: The left image shows an axial view corresponding to the mammographic craniocaudal projection. The upper-right and lower-right images show transverse and longitudinal views, respectively. (**d**) Posteroanterior view: The upper right image corresponds to the transverse ultrasound view, the lower right to the longitudinal view, and the left to the coronal view. Orientation markers are shown consistently across images: A = anterior, P = posterior, M = medial, L = lateral, S = superior, and I = inferior.

MammouS-N images were acquired with the patient in a standing position using two projections analogous to the CC and MLO mammography views. For the CC view, the ultrasound probe was positioned inferiorly with breast compression, whereas for the MLO view, it was positioned laterally (Figure 3). In the posteroanterior view (Figure 3), the patient faced the probe with a tightly compressed breast. Ultrasound gel was applied to ensure smooth contact between the probe and the breast.

Volume images were automatically transferred to a dedicated workstation. Volumetric data were acquired in the transverse plane, with slice thicknesses of 0.25–0.4 mm. The pixel spacing was 0.04 mm at the 2 cm depth and 0.06 mm at the 8 cm depth. The main viewing plane (axial) was automatically reconstructed with a uniform pixel size of 0.1 mm across all depths.

### 2.5. Image Analysis

A total of 100 DBT images, 100 MammouS-N ultrasound scans, and 100 MRI scans from 100 patients were retrospectively reviewed. Two non-masked radiologists with 24 and 11 years of experience, respectively, in breast imaging, independently evaluated all images. Interpretation was based on the structures surrounding each lesion and the lesion type. This study focused exclusively on biopsy-confirmed breast cancer. In cases with multiple biopsy-confirmed malignancies, the evaluation focused on the largest lesion. The reference standard for breast cancer diagnosis was established using all available imaging modalities, including mammography, ultrasound images obtained during biopsy, and MRI, as well as pathological results from surgical specimens. Both radiologists were informed of the approximate lesion location based on prior MRI and pathology findings. Because this study aimed to compare lesion visibility rather than detection performance, complete blinding was not feasible.

A 5-megapixel monitor and a picture archiving and communication system (Infinitt PACS^®^, Infinitt Healthcare, Seoul, Republic of Korea) were used to evaluate mammography images. MammouS-N ultrasound images were assessed using a dedicated workstation. The reviewer first examined the visibility of suspicious lesions on DBT and MammouS-N images. Subsequently, she evaluated which of the visible lesions corresponded to the biopsy-confirmed breast cancers identified on MRI. If a lesion visible on DBT or MammouS-N images did not match the lesion observed on MRI, the DBT or ultrasound images were reevaluated to locate the appropriate corresponding lesion.

Once the correct lesions were identified on DBT or MammouS-N images, visibility scores were assigned on a 6-point scale: 0 (not visible), 1 (not acceptable), 2 (poor), 3 (moderate), 4 (good), and 5 (excellent). The scores were used to assess the diagnostic value of each imaging modality.

The lesion characteristics were evaluated by consensus. For DBT, the categories included mass, asymmetry, mass with calcification, calcification only, and architectural distortion. For the MammouS-N, lesions were classified as masses or non-mass lesions. Additionally, breast density on DBT and echotexture on ultrasonography were assessed.

### 2.6. Statistical Analysis

Data were analyzed using Microsoft Excel (version 16.0; Microsoft Corporation, Redmond, WA, USA). Continuous variables comparing lesion characteristics between DBT and MammouS-N were analyzed using Student’s *t*-test. Categorical variables were compared using the chi-squared test or Fisher’s exact test, as appropriate. The Wilcoxon signed-rank test was used to compare the visibility scores of lesions identified on DBT and/or MammouS-N images. Inter-reader agreement for lesion visibility assessment was evaluated using weighted Cohen’s kappa (κ) statistics. The strength of agreement was interpreted according to the Landis and Koch guidelines: 0.21–0.40 as fair, 0.41–0.60 as moderate, 0.61–0.80 as substantial, and 0.81–1.00 as almost perfect agreement [7]. The agreement between imaging-based tumor size measurements and pathological tumor extent was evaluated using Bland–Altman analysis. To further assess potential systematic differences between each imaging modality and the pathological gold standard, the Wilcoxon signed-rank test was performed for paired comparisons. All statistical analyses were performed using the commercial R software version 4.5.2 (R Core Team, Vienna, Austria), and Stata 14.0 (Stata Corp., College Station, TX, USA). A *p*-value < 0.05 was considered statistically significant.

## 3. Results

### 3.1. Patient Characteristics

All 100 women underwent scanning using the MammouS-N system. The average scan time for acquiring six volumes of bilateral breast images was 17.1 ± 3.8 min (range: 10–30 min). Among the participants, 85 (85%) reported no pain, 12 (12%) reported mild pain, and three (3%) reported moderate pain. All participants reported experiencing less pain with the MammouS-N system than with DBT.

Table 1 summarizes the patient and tumor characteristics. Of the 100 patients, 53 (53%) were asymptomatic, 45 (45%) presented with a palpable mass, and two (2%) reported nipple discharge. Histopathologically, the study included 73 patients with invasive ductal carcinoma, 15 with ductal carcinoma in situ (DCIS), and five with DCIS and microinvasion. Additionally, three patients were diagnosed with mucinous carcinoma, two with invasive lobular carcinoma, and one patient each with adenoid cystic carcinoma and mixed invasive ductal and lobular carcinoma.

### 3.2. Mammographic and MammouS-N Characteristics

Table 2 summarizes the imaging characteristics observed using DBT and MammouS-N ultrasound. The average lesion size was 2.72 cm on DBT and 1.76 cm on MammouS-N ultrasonography. Most women (94%) showed dense breast tissue on DBT. The echotexture of the lesions on MammouS-N ultrasound was classified as homogeneous fatty in two cases, homogeneous fibroglandular in 56 cases, and heterogeneous in 40 cases.

The lesion types identified on DBT included negative findings (7%), masses (46%), masses with calcification (29%), calcifications only (15%), and architectural distortions (3%). On MammouS-N ultrasound, most lesions were classified as masses (93%), whereas 7% were non-mass lesions.

Lesion sizes were significantly larger on DBT than on MammouS-N ultrasonography across all lesion types (*p* < 0.05), except in cases with negative DBT findings.

In the subgroup of 83 patients who underwent primary surgery without neoadjuvant therapy, Bland–Altman analysis showed no significant mean difference between DBT measurements and pathological tumor extent (mean difference, −0.24 cm; *p* = 0.18), although wide limits of agreement were observed. In contrast, MammouS-N measurements significantly underestimated the pathological tumor extent, with a mean difference of 0.77 cm (*p* < 0.0001). Wilcoxon signed-rank tests confirmed that DBT measurements did not differ significantly from pathology (*p* = 0.61), whereas MammouS-N measurements were significantly smaller than pathological size (*p* < 0.0001).

### 3.3. Breast Cancer Visibility and Influencing Factors

Table 3 and Table 4 present the visibility scores for breast cancer on DBT and MammouS-N, as assessed by the two reviewers. For Reviewer 1, 26 cases had higher visibility scores with MammouS-N, seven had higher scores with DBT, and 67 had equal scores for both. MammouS-N demonstrated significantly higher visibility scores compared to DBT (z = −3.234, *p* = 0.001). Of the 26 higher visibility scores, 12 were not detected on DBT, and 14 showed a low visibility score of 1 on DBT. According to Reviewer 2, 27 cases had higher scores with MammouS-N, 28 had higher scores with DBT, and 45 had equal scores for both. There was no significant difference in visibility between the two modalities for this reviewer (z = −0.040, *p* = 0.968), indicating no clear preference. Of the 28 higher visibility scores, seven were not detected on DBT. Inter-reader agreement for lesion visibility was moderate for both modalities. The weighted kappa value was 0.48 (95% CI, 0.36–0.61) for DBT and 0.41 (95% CI, 0.27–0.55) for MammouS-N.

**Figure 4:**
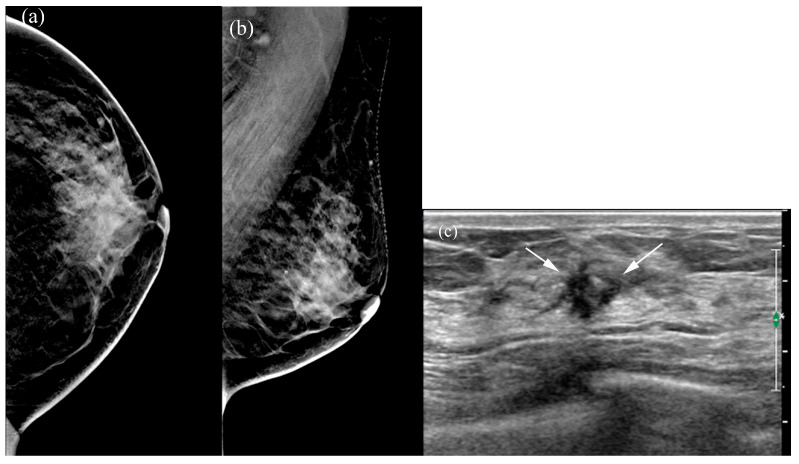

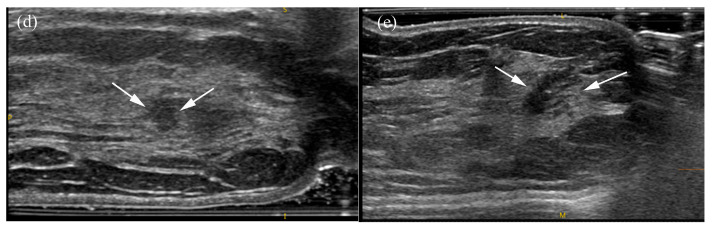
A 66-year-old female patient with screen-detected invasive lobular and ductal carcinoma: (**a**,**b**) The malignancy is not visualized on digital breast tomosynthesis (DBT). (**c**) Hand-held ultrasound reveals a spiculated, irregular, heterogeneous hypoechoic mass (arrows) in the left 12 o’clock area. (**d**,**e**) MammouS-N transverse images in craniocaudal (**d**) and mediolateral oblique (**e**) views demonstrate an indistinct, irregular, heterogeneous hypoechoic mass (arrows) in the central upper area of the left breast. S = superior, I = inferior, and M = medial.

**Figure 5:**
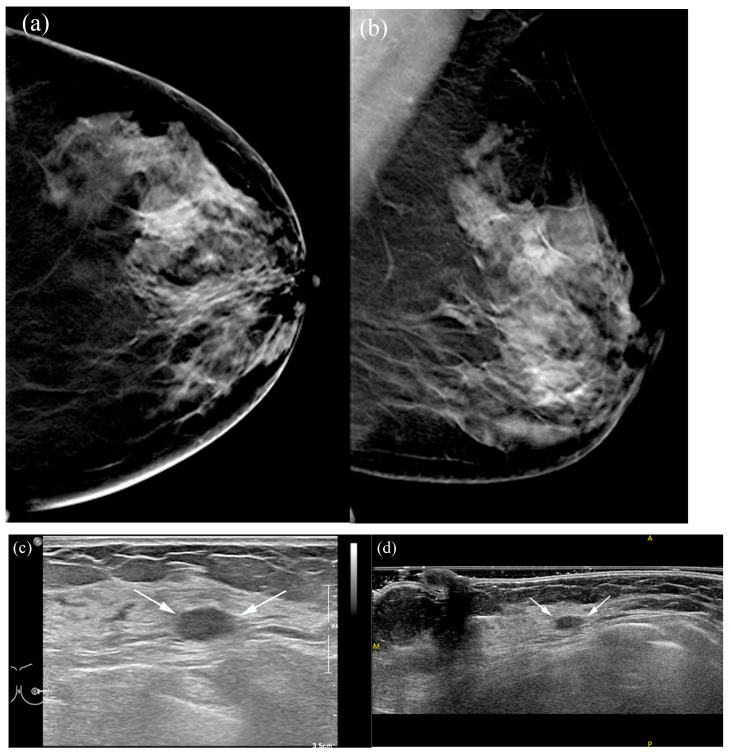
A 50-year-old female patient with left-sided bloody nipple discharge: (**a**,**b**) The malignancy is not visualized on digital breast tomosynthesis (DBT). (**c**) Hand-held ultrasound reveals an indistinct, oval hypoechoic mass (arrows) in the left 3 o’clock area. (**d**) MammouS-N transverse posteroanterior image shows an indistinct, oval hypoechoic mass (arrows) in the left 3 o’clock area (A, anterior; P, posterior; M, medial). Surgical pathology confirmed microinvasive secretory carcinoma.

**Figure 6:**
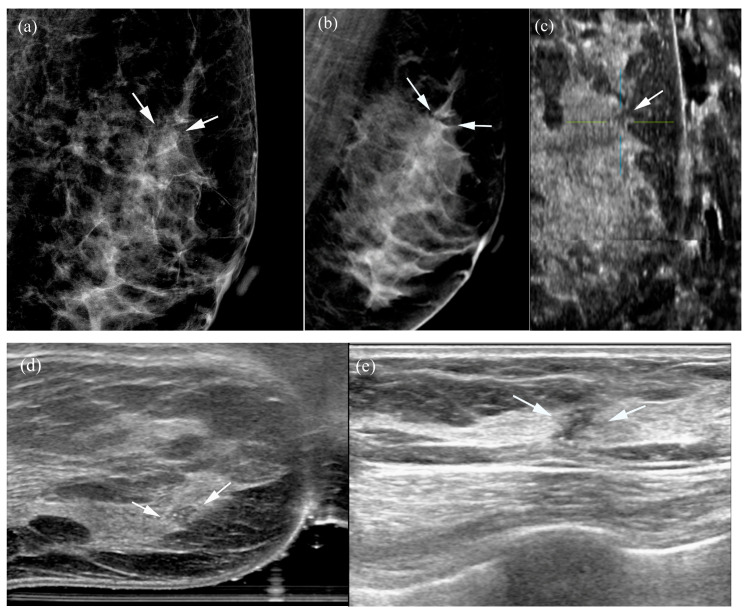
A 56-year-old woman with ductal carcinoma in situ: (**a**,**b**) Two-dimensional and three-dimensional left mediolateral oblique mammograms demonstrate grouped pleomorphic microcalcifications and associated architectural distortion (arrows). (**c**–**e**) MammouS-N sagittal (**c**) and transverse (**d**) images of the left mediolateral oblique view reveal an irregular, angular hypoechoic mass containing internal echogenic foci (crosshairs, arrows), representing microcalcifications. These findings align with the features observed on the corresponding handheld ultrasound image (**e**).

Table 5 summarizes the factors influencing lesion visibility on DBT and MammouS-N images. Lesions that were better visualized on MammouS-N included masses obscured on DBT and non-calcified lesions (*p* ≤ 0.005) (Figure 4 and Figure 5). Reviewer 2’s assessment showed that larger lesions tended to be better visualized using DBT. No significant associations were observed between lesion visibility and factors such as patient age, mammographic density, lesion size on MammouS-N, echotexture, or lesion type (Figure 6).

## 4. Discussion

This study compared the visibility of malignant breast lesions on DBT and MammouS-N, a standing ABUS, in a population predominantly composed of women with dense breast tissue (94%). MammouS-N demonstrated superior visibility in 26 cases according to Reviewer 1 and in 27 cases according to Reviewer 2, with the difference reaching statistical significance for Reviewer 1. MammouS-N detects malignancy in at least seven cases of malignancy that DBT missed due to the obscured dense parenchyma.

Women with dense breast tissue face a well-established dual challenge during breast cancer screening: a modestly elevated risk of developing breast cancer and reduced sensitivity to mammography owing to tissue-masking effects [8,9]. Although DBT offers notable improvements over conventional mammography in the imaging of dense breast tissue, tissue superimposition remains a limiting factor for lesion visibility [4].

Our findings align with those of previous studies, highlighting the complementary value of ultrasound-based screening in women with dense breasts. A prospective comparative trial demonstrated that ultrasound detected additional cancers not identified by DBT in dense breast tissue with negative mammography findings, suggesting that certain lesions were visible on ultrasound but not on DBT [10]. Similarly, when ABUS is used as a supplemental screening tool, cancer detection rates improve beyond those of DBT alone, with some invasive cancers being exclusively detected by ABUS [8,11].

Recent comparative studies support these findings. Aribal et al. demonstrated that ABUS and DBT achieved comparable cancer detection rates (7.5/1000 screenings) when used as supplemental screening tools, suggesting an equivalence between the two modalities in terms of cancer detection efficacy [12]. Additionally, a systematic review and meta-analysis by Zhang et al. confirmed that ABUS significantly improved cancer detection in women, reporting a pooled sensitivity of 0.88 (95% CI: 0.73–0.95) and specificity of 0.93 (95% CI: 0.82–0.97) [13]. MammouS-N appears to offer similar advantages, particularly by providing additional information on small, noncalcified lesions that may be obscured by the dense parenchyma on DBT.

Lesion characteristics strongly influenced the visibility differences between the two modalities. Non-calcified lesions received high visibility scores on both DBT and MammouS-N, according to both reviewers. However, DBT demonstrated superior performance in detecting calcified lesions, accounting for 44% of the cases in this study. This result was expected, as tomosynthesis is particularly effective for detecting calcifications owing to its high-contrast appearance, whereas ultrasound generally has limited sensitivity for microcalcifications unless they are associated with a mass or non-mass lesion [14,15]. On MammouS-N, 93% of the lesions were classified as masses, and only 7% were classified as non-mass lesions. This distribution suggests that MammouS-N may be particularly valuable for enhancing the detection and characterization of mass lesions, particularly smaller lesions without associated calcifications, which may be more challenging to identify using DBT.

An important finding of this study was the difference in the measured lesion sizes between the two modalities, with DBT showing a larger average lesion size (2.72 cm) than MammouS-N (1.76 cm). This size difference was consistently observed across all lesion types detected using both modalities and may be attributed to several factors. On mammography, regions of calcification or spiculated margins often appear larger than the actual invasive components. This phenomenon is supported by findings from the STORM-2 trial, which reported that DBT frequently overestimates tumor size, particularly in women with dense breast tissue [16]. This overestimation occurs because DBT captures both DCIS components and invasive cancer, whereas pathological measurements typically reflect only the invasive component [16]. In line with these observations, our findings indicate that DBT measurements were, on average, comparable to pathological tumor extent, albeit with substantial individual variability. In contrast, MammouS-N systematically underestimated tumor size relative to pathology. This discrepancy likely reflects an inherent limitation of ultrasound-based modalities, which primarily visualize the invasive component of a tumor. In contrast, pathological extent often includes surrounding in situ components and microscopic spread, which are typically better captured by DBT or MRI than by ultrasound [17,18]. These discrepancies have important implications for treatment planning and surgical decision-making. Overestimation may lead to more extensive surgery, whereas underestimation may risk incomplete excision. Thus, understanding modality-specific tendencies in lesion size measurement is crucial when interpreting results from MammouS-N.

These findings have substantial clinical implications for breast cancer screening protocols in women with dense breast tissue. Dense breast tissue not only increases cancer risk but also poses diagnostic challenges, reinforcing the need for multimodal imaging strategies [19]. Integrating MammouS-N into screening protocols may improve cancer detection in this population, given its high sensitivity and visibility compared with DBT, along with the added benefits of radiation-free imaging and less pain. In terms of workflow, the MammouS-N examination can be performed in a standing position, potentially allowing integration into existing DBT screening suites. Although the examination time (up to 30 min) may initially appear long, process optimization and operator experience are expected to reduce acquisition time. The cost implications remain uncertain and will depend on adoption scale and reimbursement policies, but MammouS-N could reduce operator workload compared with handheld ultrasound.

This study has some limitations that should be considered when interpreting the findings. First, the relatively small sample size and the fact that most biopsies were ultrasound-guided may limit the generalizability of our findings to settings with different diagnostic workflows. Additionally, as a tertiary center study, our cohort may reflect a selection bias toward patients whose lesions were already localized by prior imaging. Second, the image analysis was performed by two radiologists with prior knowledge of lesion locations based on MRI and pathology. While this approach was necessary to evaluate comparative lesion conspicuity, it introduces expectation bias and does not reflect real-world diagnostic conditions. Therefore, our findings should be interpreted as an evaluation of visibility enhancement rather than independent diagnostic efficacy. Third, 94% of our study population had dense breast tissue. While this aligns with the high prevalence of dense breasts in Korean women [20], it may introduce selection bias. Therefore, our findings should be interpreted with caution when applied to populations with lower breast density. Fourth, operator dependency is an important consideration for MammouS-N implementation because image quality can vary substantially depending on the operator’s experience and technique. Skaane et al. demonstrated that ABUS interpretation was significantly influenced by reader experience, with area under the curve values of 0.592–0.904, depending on the reader’s level of expertise [21]. This learning curve may affect the visibility and detection rates of lesions in clinical practice. Fifth, a direct quantitative comparison between HHUS and MammouS-N was not performed because HHUS images were available only as representative static captures rather than comprehensive volume data. This limited the ability of independent reviewers to retrospectively apply the same visibility scoring system. Future prospective trials utilizing standardized evaluation criteria for both modalities are warranted to further clarify their relative clinical utility. Finally, the present study did not evaluate intra-reader variability, which is a critical factor in ABUS interpretation. Mendelson and Berg emphasized the importance of standardized training protocols and quality assurance measures to ensure the successful implementation of ABUS in clinical settings [22]. Future studies should incorporate blinded assessments and explicitly evaluate reader variability to better reflect real-world diagnostic conditions.

## 5. Conclusions

In conclusion, this study demonstrated that MammouS-N holds promise as a complementary imaging modality to DBT in women with dense breast tissue, particularly for the detection of non-calcified lesions. Although DBT has clear advantages in identifying calcified lesions, MammouS-N offers notable benefits, including radiation-free imaging and superior soft-tissue contrast. Given its automated, reproducible imaging and the ability to integrate into DBT workflows, MammouS-N could serve as a practical adjunct to DBT, particularly in populations where supplemental ultrasound screening is recommended. Future studies should explore integrated imaging protocols that combine the strengths of both modalities with the goal of improving diagnostic accuracy and clinical outcomes, especially in high-risk populations such as women with dense breast tissue.

## Figures and Tables

**Table 1:** Patient and tumor characteristics (*n* = 100).

Variable	Data
Age (years)	51.6 ± 9.3 (range, 26–76)
Symptoms	
Asymptomatic	53 (53.0)
Palpable mass	45 (45.0)
Nipple discharge	2 (2.0)
Histologic type	
Invasive ductal carcinoma	73 (73.0)
DCIS	15 (15.0)
DCIS with microinvasion	5 (5.0)
Mucinous	3 (3.0)
Invasive lobular	2 (2.0)
Adenoid cystic carcinoma	1 (1.0)
Mixed invasive ductal and lobular carcinoma	1 (1.0)

Data are presented as the number of patients, with percentages in parentheses, or as mean ± standard deviation. DCIS = ductal carcinoma in situ.

**Table 2:** Digital breast tomosynthesis and MammouS-N characteristics (*n* = 100).

DBT		MammouS-N		*p*-Value
Size (cm)	2.72 ± 1.95 (range: 0–9.4)	MammouS-N size (cm)	1.76 ± 0.82 (range: 0.7–4.1)	0.007
Density *		Echotexture		
b	6 (6.0)	a	2 (2.0)	
c	54 (54.0)	b	56 (56.0)	
d	40 (40.0)	c	42 (42.0)	
Lesion type		Lesion type		
Negative	7 (7.0)	Mass	93 (93.0)	
Mass	46 (46.0)	Non-mass lesion	7 (7.0)	
Mass with calcifications	29 (29.0)			
Calcifications	15 (15.0)			
Architectural distortion	3 (3.0)			
Size according to DBT lesion type (cm)	DBT size (cm)	MammouS-N size (cm)		
Negative	0		1.06 ± 0.3	<0.001
Mass	2.47 ± 1.54		1.78 ± 0.78	0.007
Mass with calcifications	2.76 ± 1.84		1.87 ± 0.8	0.020
Calcifications	4.09 ± 2.53		1.78 ± 0.97	0.002
Architectural distortion	3.55 ± 1.78		1.53 ± 0.79	0.084
Size according to MammouS-N lesion type (cm)	DBT size (cm)	MammouS-N size (cm)		
Mass	2.67 ± 1.85		1.8 ± 0.978	<0.001
Non-mass lesion	3.09 ± 2.61		1.4 ± 0.73	0.045

Data are presented as the number of patients, with percentages in parentheses, or as mean ± standard deviation. * Breast density was assessed according to the Breast Imaging Reporting and Data System (BI-RADS). DBT = digital breast tomosynthesis. Breast density was assessed according to the Breast Imaging Reporting and Data System (BI-RADS): b, scattered fibroglandular density; c, heterogeneously dense; d, extremely dense. Echotexture: a, homogeneous background echotexture–fat; b, homogeneous background echotexture–fibroglandular; c, heterogeneous background echotexture.

**Table 3:** Visibility scores on DBT and MammouS-N by Reviewer 1.

DBT Visibility Score	MammouS-N Visibility Score						
	0	1	2	3	4	5	Total
0	1	9 *	0	0	0	3 *	13
1	0	15	0	0	0	14 *	29
2	0	0	0	0	0	0	0
3	0	0	0	1	0	0	1
4	0	1	0	0	0	0	1
5	0	6	0	0	0	50	56
Total	1	31	0	1	0	67	100

* Indicates cases where lesions were better visualized on MammouS-N than on DBT. DBT = digital breast tomosynthesis.

**Table 4:** Visibility scores on DBT and MammouS-N by Reviewer 2.

DBT Visibility Score	MammouS-N Visibility Score						
	0	1	2	3	4	5	Total
0	0	2	2	0	1	2	7 *
1	0	0	2	1	0	2	5 *
2	0	0	0	2	0	3	5 *
3	0	0	3	1	0	5 *	9
4	0	2	2	1	3	5 *	13
5	0	3	3	3	11	41	61
Total	0	7	12	8	15	58	100

* Indicates cases where lesions were better visualized on MammouS-N than on DBT. DBT = digital breast tomosynthesis.

**Table 5:** Factors affecting lesion visibility: DBT vs. MammouS-N.

Factors	Modality for Better Visualization (Reviewer 1)			Modality for Better Visualization (Reviewer 2)		
	DBT * (*n* = 74)	MammouS-N (*n* = 26)	*p*-Value	DBT * (*n* = 73)	MammouS-N (*n* = 27)	*p*-Value
Age (years)	51.4 ± 9.8	51.6 ± 7.8	0.972	51.8 ± 9.6	50.1 ± 8.7	0.663
Size on DBT	2.91 ± 2.0	2.1 ± 1.7	0.055	3.0 ± 2.0	1.87 ± 1.8	0.011
Density ^†^			0.295			0.239
b	6 (8.1)	0 (0.0)		6 (8.2)	0 (0.0)	
c	40 (54.1)	14 (53.8)		40 (54.8)	14 (51.9)	
d	28 (37.8)	12 (46.2)		27 (37.0)	13 (48.1)	
Lesion type on DBT			0.005			<0.001
Negative	1 (1.4)	6 (23.1)		0 (0.0)	7 (25.9)	
Mass	36 (48.6)	10 (38.5)		34 (46.6)	12 (44.4)	
Mass with calcifications	24 (32.4)	5 (19.2)		24 (32.9)	5 (18.5)	
Calcifications	11 (14.9)	4 (15.4)		15 (20.5)	0 (0.0)	
Architectural distortion	2 (2.7)	1 (3.8)		0 (0.0)	3 (11.1)	
Size on MammouS-N	1.8 ± 0.8	1.7 ± 0.9	0.665	1.8 ± 0.8	1.6 ± 0.7	0.109
Echotexture			0.616			0.759
a	1 (1.4)	1 (3.8)		1 (1.4)	1 (3.7)	
b	43 (58.1)	13 (50.0)		41 (56.2)	15 (55.6)	
c	30 (40.5)	12 (46.2)		31 (42.5)	11 (40.7)	
Lesion type on MammouS-N			1			0.590
Mass	69 (93.2)	24 (92.3)		69 (94.5)	24 (88.9)	
Non-mass lesion	5 (6.8)	2 (7.7)		4 (5.5)	3 (11.1)	

* Indicates lesions that were better or equally visualized on DBT than on MammouS-N. ^†^ Breast density was assessed according to the Breast Imaging Reporting and Data System (BI-RADS). DBT = digital breast tomosynthesis. Breast density was assessed according to the Breast Imaging Reporting and Data System (BI-RADS): b, scattered fibroglandular density; c, heterogeneously dense; d, extremely dense. Echotexture: a, homogeneous background echotexture–fat; b, homogeneous background echotexture–fibroglandular; c, heterogeneous background echotexture.

## Data Availability

The data presented in this study are available upon reasonable request from the corresponding author. The data are not publicly available due to privacy and ethical restrictions.

## Abbreviations

The following abbreviations are used in this manuscript:

ABUS	automated breast ultrasound system
CC	craniocaudal
3D	3 dimensional
DCIS	ductal carcinoma in situ
DBT	digital breast tomosynthesis
MLO	mediolateral oblique
MRI	magnetic resonance imaging
